# A patient with lumbar disc herniation complicates cauda equina syndrome after epidural steroid injection: A case report

**DOI:** 10.1097/MD.0000000000048739

**Published:** 2026-05-15

**Authors:** Chuangye Zhang, Xin Xin, Pengpeng Qi, Tonghao Sheng, Zhongyuan Liu, Lu Yang, Xinzhen Che, Nianhu Li, Jingguo Wu

**Affiliations:** a Department of Spine and Spinal Cord, Affiliated Hospital, Shandong University of Traditional Chinese Medicine, Jinan, China.

**Keywords:** cauda equina syndrome, epidural injection, intervertebral disc displacement, paraplegia

## Abstract

**Rationale:**

While epidural steroid injections (ESI) are a routine component of lumbar disc herniation management, the procedure carries inherent risks. Certain clinical presentations may signal an elevated risk for catastrophic complications, such as cauda equina syndrome. This report examines the significance of severe positional pain as a pre-procedural warning sign.

**Patient concerns:**

We present the case of a 65-year-old male who exhibited a critical physical sign: severe low back pain prohibiting a supine position. Despite this red flag, an interlaminar ESI was administered at the L2–3 level without prior definitive magnetic resonance imaging (MRI) evaluation.

**Diagnoses:**

Following the procedure, the patient progressed to acute paraplegia. Emergency MRI identified a massive L2–3 disc extrusion with near-total spinal canal occlusion, confirming a diagnosis of cauda equina syndrome.

**Interventions:**

The patient underwent emergency surgical decompression. Prior to this, he had received the interlaminar ESI which preceded the neurological decline.

**Outcomes:**

At the 4-month postoperative follow-up, the patient demonstrated partial neurological recovery, regaining the ability to ambulate short distances with the aid of a walker.

**Lessons:**

The inability to assume a supine position due to pain should be regarded as a marker for severe spinal stenosis or a large disc herniation. Proceeding with ESI without advanced imaging in such contexts poses an unacceptable risk of precipitating permanent neurological deficit. Comprehensive imaging is mandatory; if pain prevents MRI acquisition, alternative diagnostic strategies must be prioritized over blind intervention.

## 1. Introduction

Lumbar disc herniation (LDH) often leads to low back pain (LBP) and radiculopathy. Lumbar disc herniation usually resolves spontaneously over time, with a natural resorption rate of >60%.^[1]^ Therefore, the consensus for the treatment of patients with LDH is to begin conservative treatment followed by surgical intervention in patients for whom conservative treatment is ineffective.^[2]^ One conservative treatment for LDH is epidural steroid injection (ESI). ESI involves injecting hormones and local anesthetics into the epidural space. Numerous reports and extensive reviews have confirmed the role of ESI in the relief of LBP and radiculopathy pain.^[3]^

Cauda equina syndrome is a rare complication of ESI. The syndrome results from sacral nerve root injury and is characterized by varying degrees of bladder and bowel dysfunction, loss of sensation in the perineum, and motor weakness in the lower extremities.^[4]^ Although complications such as vascular embolism, bone loss, hyperglycemia, infection, vascular injury, epidural hematoma, nerve injury, and paraplegia have been reported after epidural steroid injections, acute neurologic symptoms have been rarely reported.^[5]^ In May 2025, Shandong Hospital of Traditional Chinese Medicine admitted a patient with LDH complicated with cauda equina syndrome after interlaminar epidural closure and it is reported as follows. This case underscores the need to recognize severe positional pain as a contraindication for ESI without prior imaging, which is the key lesson this report aims to illustrate.^[6]^

## 2. Case report

A 65-year-old male was admitted in May 2025 with a 20-day history of LBP accompanied by radiating pain and numbness along the left lower limb, which acutely worsened the day before admission. Regarding the patient’s lower back pain, only bed rest was adopted as the treatment approach, and no systematic therapeutic measures were implemented. The symptoms began spontaneously without a clear cause.

### 2.1. Clinical presentation

Upon examination, the patient reported sharp pain originating from the lumbar region, radiating along the left buttock, posterolateral thigh, and lateral calf to the ankle, with numbness following a similar distribution. Although the sensation in the left foot remained intact, the right foot exhibited numbness. The pain impaired weight-bearing and lying flat and was partially relieved only in a seated position. Neurological assessment showed sensory deficits in the left lower limb, particularly over the anterior thigh. Both the left knee and ankle reflexes were graded 3+. The Babinski sign was absent. The straight leg raise test was positive on the left side at 30°, and the reinforcing test was positive. The right lower limb showed no motor or sensory impairments. There were no findings suggestive of cauda equina syndrome, such as urinary retention, saddle anesthesia, or decreased anal tone. The patient had no significant medical history of hypertension, diabetes, or prior spinal disorders.

### 2.2. Initial management

After admission, the patient was administered flurbiprofen ester injection (100 mg IV drip), parecoxib sodium injection (40 mg IV drip), ketorolac tromethamine injection (30 mg IM), buclizine hydrochloride injection (100 mg IM), dexamethasone sodium phosphate injection (10 mg IV drip), and mannitol injection (125 mL IV drip) to reduce swelling and pain.

### 2.3. Diagnostic challenge and decision for ESI

After 3 days of conservative treatment, the patient was still unable to lie down for an magnetic resonance imaging (MRI) due to pain. The patient strongly requested that pain relief be prioritized before imaging. Conventional analgesics were ineffective; therefore, we invited an anesthesiologist to assist in the consultation. After completing the preoperative anesthesia examination and assessment, and following a discussion of risks, we decided to perform an epidural steroid injection (ESI).

### 2.4. ESI procedure

On the fourth day after admission, the patient underwent an interlaminar epidural steroid injection at the L2–3 level in the operating room. The medications used were 1% lidocaine hydrochloride (5 mL), 0.5% ropivacaine hydrochloride (3 mL) for the block test, and dexamethasone sodium phosphate (5 mg). The puncture was performed at the L2–3 interspinous space. No immediate complications, cerebrospinal fluid leakage, or abnormal sensations were observed. The patient returned to the ward with effective analgesia.

### 2.5. Post-procedural deterioration and diagnosis

**Figure 1.**
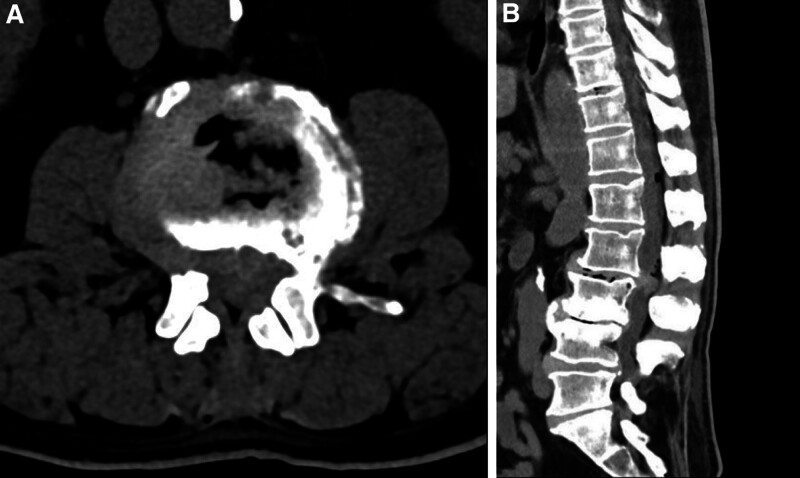
(A) Axial and (B) sagittal CT images showing disc herniation with bilateral radicular stenosis at L2/3, L4/5, and L5/S1, and stenosis of the L2/3 spinal canal. Multiple intervertebral disc pneumatization. CT = computed tomography.

**Figure 2.**
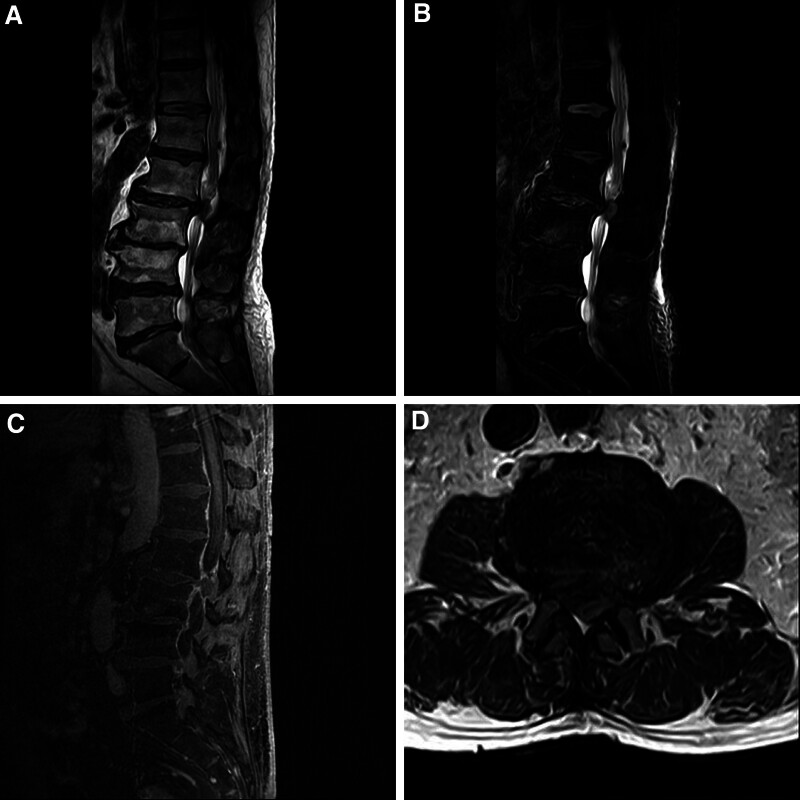
(A) T1W sagittal image, (B) T2W sagittal image, (C) enhanced sagittal image, and (D) T2W axial image showing limited abnormal signal in the spinal canal at the L2/3 level, with consideration of nucleus pulposus prolapse of the intervertebral discs. Mild posterior slippage of L2 and L3 vertebral bodies was demonstrated. Extremely low signal was seen in the lumbar spinal canal in all sequences, and the possibility of a little gas was considered.

However, 10 hours after the ESI procedure, the patient reported worsening swelling and numbness in both lower limbs with an “ant sensation,” grade 0 muscle strength below both knees, decreased sensation, inability to move both calves, numbness in the perineal area, and difficulty in urinating, which was treated with urinary catheter insertion. Cauda equina syndrome (CES) was highly suspected. Emergency MRI and a complementary non-contrast computed tomography (CT) scan were performed (Figs. 1 and 2), revealing a massive L2–3 disc herniation causing severe spinal canal compromise and cauda equina syndrome, with evidence suggesting the presence of epidural air. Additionally, imaging features such as disc space vacuum phenomenon were noted, consistent with underlying lumbar spondylosis.

### 2.6. Surgical intervention

**Figure 3.**
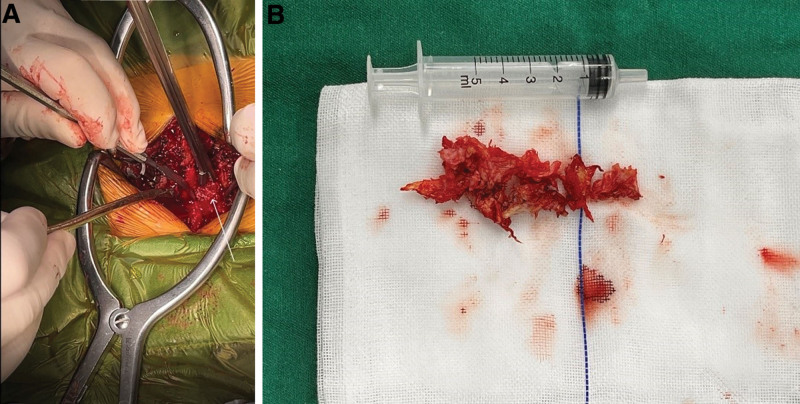
(A) Intraoperative view of the extracted herniated nucleus pulposus, (B) extracted nucleus pulposus tissue.

An emergency posterior lumbar interbody fusion (PLIF) with dural incision and exploration was performed 10 hours after the onset of paraplegia. The surgeon removed the L2 bilateral vertebral plates, the L2 inferior articular process, and part of the L3 superior articular process, excised part of the ligamentum flavum, and explored the spinal canal. A large amount of herniated nucleus pulposus tissue was found compressing the ventral and left dorsal aspects of the dura mater and the L3 nerve root. The herniated material was removed, decompressing the dural sac and nerve root (Fig. 3). The dura was closed, and the spine was stabilized with instrumentation and bone graft.

### 2.7. Postoperative course and follow-up

After surgery, the patient’s lower limbs were less swollen and numb, but paralysis was not relieved. The bilateral skeletal muscle strength was grade 3+, quadriceps muscle strength was grade 3, tibialis anterior, extensor digitorum longus, and calf triceps muscle strength was grade 0. There was decreased sensation below the left knee and right ankle and numbness in the perineal area. Her ability to perform activities of daily living (ADLs) was scored 45 points, with moderate dysfunction.

Postoperative management included bed rest, dehydration, hormone shock, and neurotrophic therapy, alongside a multimodal rehabilitation program comprising physiotherapy, acupuncture, intermediate-frequency pulsed electricity therapy, and hyperbaric oxygen therapy.

The patient remained hospitalized for 8 weeks postoperatively. At discharge, bilateral lower limb numbness had significantly diminished, with light touch and proprioception restored in the lower legs. Motor assessment revealed bilateral hip girdle muscle strength grade 3+, quadriceps grade 3, and tibialis anterior, extensor hallucis longus, and triceps surae muscle grade 2. Muscle tone was normal.

**Figure 4.**
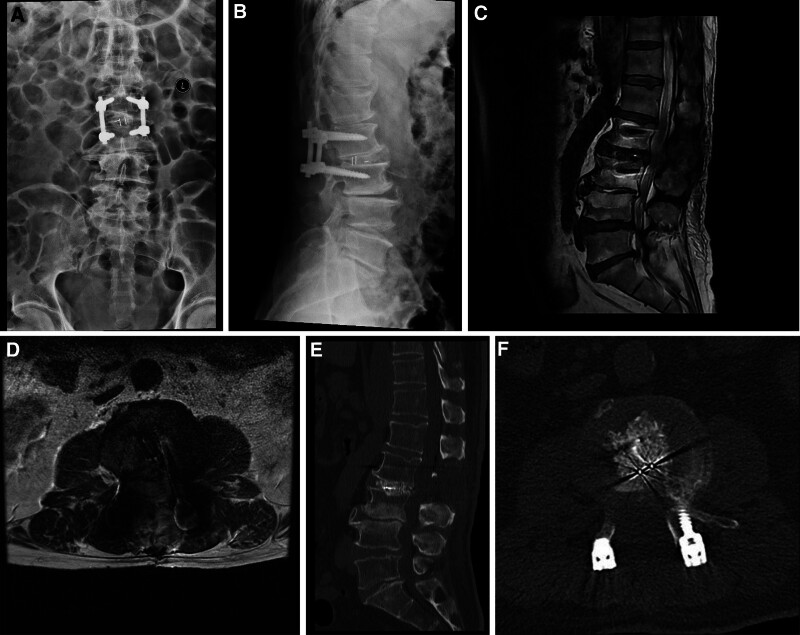
(A, B) Frontal and lateral DR of the lumbar spine, (C) T2W sagittal image, (D) T2W axial image, and (E) sagittal and (F) axial CT images demonstrating that there was no longer a buildup of gas in the spinal canal, and that there was adequate decompression of the spinal canal. CT = computed tomography, DR = digital radiography.

The patient was then transferred to a local rehabilitation facility. Follow-up radiographic imaging (Fig. 4) demonstrated successful surgical decompression with no evidence of residual gas or stenosis. A structured telephone follow-up at 4 months postoperatively demonstrated continued functional improvement, notably the ability to ambulate short distances with a walker.

## 3. Discussion

Cauda equina syndrome is an acute neuropathy that causes varying degrees of urinary and fecal incontinence, loss of local sensation in the perineum, and varying degrees of leg weakness by affecting smaller or autonomic nerve fibers.^[7]^ This is a very rare complication of ESI. Severe L2–3 LDH may result in severe pain and paralysis. Presumably the patient avoids paralysis by maintaining spaciousness of the spinal canal by keeping the lumbar spine in forward flexion.^[8]^ Therefore, the patient may have refused to adopt a lying position with the spine extended because of the severe pain he felt. This patient developed paraplegia after ESI and his condition rapidly deteriorated. The relationship between cauda equina syndrome and ESI in this patient was unclear. However, there may be several reasons for CES in this patient.

First, CES may have been due to the rapid increase in pressure in the epidural space when the drugs were injected during the maneuver. According to a study by Usubiaga et al,^[9]^ when 10 mL of 2% lidocaine is injected into the epidural space, the intraluminal pressure can increase from −10 cm H_2_O to a maximum of 65 cm H_2_O. This is especially true in elderly patients,^[10]^ where the epidural pressure is even higher and the pressure level can be maintained for as long as 2 minutes after the injection of the drug. The volume of the epidural space in our patient was too small to tolerate pain without adopting a lumbar forward flexion position. Therefore, we believe that the pressure effect produced by the drug injection into the epidural space is stronger. In elderly patients, who are expected to have high epidural pressure due to severe disc herniation, small amounts of medication should be injected as slowly as possible.

Another report on pneumatic ridge syndrome (PR) by Khai et al^[11]^ suggested that an epidural air cavity should be considered as a potential etiologic factor in patients presenting with CES after ESI. Epidural air spaces are usually asymptomatic because air tends to not diffuse and is naturally absorbed. Our patient developed CES symptoms after ESI was performed at the L2/L3 disc level. CT and MRI of our patient after ESI showed very low signals in various sequences within the lumbar spinal canal, suggesting the presence of an epidural air cavity. Postoperative review of MRI and CT showed that there was no longer pneumatic gas accumulation compressing the dura, resulting in spinal canal stenosis, moderate compression of the cerebrospinal fluid space, and some cauda equina nerve root aggregates. The treatment of PR depends on a variety of factors such as the underlying etiology, severity of the symptoms, and degree of compression of the spinal cord.^[12]^ PR is a rare condition requiring careful evaluation and appropriate management. Despite its low prevalence, clinical vigilance remains critical for facilitating early detection and timely intervention.

Third, changes in position after pain relief may have exacerbated the condition. There have been case reports suggesting that CES develops according to a similar mechanism^[13]^ anterior lumbar flexion position aggravates LDH by exerting pressure on the disc, but the anterior lumbar flexion position increases the volume of the spinal canal.^[8]^ As previously mentioned, the patient already had severe LDH and resulting spinal cord compression syndrome, but her position may have increased the diameter of the spinal canal through lumbar forward flexion, thereby reducing the pressure on the dural sac.^[14]^ However, after undergoing transforaminal interbody epidural closure (ESI), the patient is able to lie supine and can therefore extend the spine posteriorly. At this point, the volume of the spinal canal decreases and strong compression of the dural sac is expected, which immediately triggers CES.

Beyond confirming the known risks of ESI, the distinctive contribution of this case is its emphasis on a specific pre-procedural clinical sign as a decisive contraindication. The detailed chronology of events demonstrates that the inability to lie supine due to pain is not merely a logistical hurdle for imaging, but a cardinal sign of mechanical instability and critical stenosis. This nuanced insight, highlighting the peril of overlooking functional intolerance, constitutes the core novel message of our report and should be integrated into pre-procedural risk assessment protocols.

Prior to posterior lumbar interbody fusion (PLIF) surgery, preoperative magnetic resonance imaging (MRI) evaluation is important to determine the patient’s neurologic status and etiology. However, as was the case in this case, treating the patient may be challenging if the patient is unable to change positions and imaging is unavailable. If magnetic resonance imaging equipment capable of performing examinations in different positions, such as sitting or standing, is not available,^[15,16]^ the cause and severity of pain and the risk of surgery should be determined by reviewing the patient’s history and performing a neurologic examination. Prior to imaging, assessment of whether a higher grade of analgesic medication, such as gabapentinoids or morphine-based drugs^[17]^ should be used instead of ESI.

## 4. Conclusion

In conclusion, performing ESI without prior definitive MRI is a high-risk procedure in patients with severe positional pain, as it may precipitate CES in the setting of underlying critical spinal stenosis. A thorough imaging evaluation is imperative before intervention, even in the face of significant pain-related diagnostic challenges. We performed an ESI without an accurate initial assessment of the patient’s condition, and the patient developed paraplegia after surgery. Even when we performed timely PLIF + dural incision exploratory surgery, the patient’s paraplegia did not improve. It is crucial to accurately assess a patient’s condition, develop a correct treatment plan, and adopt a multidisciplinary treatment model before performing this procedure. It is also important to explain the risks and possible complications of surgery to prepare for possible serious complications. However, even with better measures, complications can still occur postoperatively. Once a complication occurs, rapid identification of the cause and timely response can have a significant impact on patient recovery.

## 5. Merits and constraints

The principal strength of this case study is its comprehensive diagnostic documentation, which captures the rapid progression from intervention to a rare but catastrophic complication. The detailed clinical timeline, supported by serial imaging and neurological assessments, provided a clear clinicopathological correlation. Furthermore, the report benefits from a structured medium-term follow-up that objectively documents the patient’s neurological recovery. However, these findings are subject to the inherent limitations of single case reports. Although it offers valuable mechanistic insight and a critical clinical lesson, its generalizability is constrained, and the findings warrant validation in larger prospective studies.

## Author contributions

**Conceptualization:** Chuangye Zhang, Jingguo Wu, Xin Xin, Pengpeng Qi, Nianhu Li.

**Data curation:** Chuangye Zhang, Nianhu Li.

**Formal analysis:** Chuangye Zhang, Xin Xin.

**Investigation:** Chuangye Zhang, Jingguo Wu, Lu Yang, Nianhu Li.

**Methodology:** Chuangye Zhang, Jingguo Wu, Pengpeng Qi, Lu Yang, Nianhu Li.

**Project administration:** Nianhu Li.

**Software:** Tonghao Sheng.

**Supervision:** Pengpeng Qi, Lu Yang, Xinzhen Che, Nianhu Li.

**Validation:** Chuangye Zhang, Pengpeng Qi, Tonghao Sheng, Zhongyuan Liu, Lu Yang, Xinzhen Che, Nianhu Li.

**Visualization:** Jingguo Wu, Xin Xin, Tonghao Sheng, Zhongyuan Liu, Lu Yang, Nianhu Li.

**Writing – original draft:** Chuangye Zhang.

**Writing – review & editing:** Jingguo Wu, Nianhu Li.
